# Assessing Potential Impact of Bt Eggplants on Non-Target Arthropods in the Philippines

**DOI:** 10.1371/journal.pone.0165190

**Published:** 2016-10-31

**Authors:** Mario V. Navasero, Randolph N. Candano, Desiree M. Hautea, Randy A. Hautea, Frank A. Shotkoski, Anthony M. Shelton

**Affiliations:** 1 National Crop Protection Center/CPC, College of Agriculture, University of the Philippines Los Baños, College, Laguna, Philippines; 2 Institute of Plant Breeding/CSC, College of Agriculture, University of the Philippines Los Baños, College, Laguna, Philippines; 3 International Service for the Acquisition of Agri-Biotech Applications, Los Baños, Laguna, Philippines; 4 International Programs, Cornell University, Ithaca, New York, United States of America; 5 Department of Entomology, Cornell/NYSAES, Geneva, New York, United States of America; Institut Sophia Agrobiotech, FRANCE

## Abstract

Studies on potential adverse effects of genetically engineered crops are part of an environmental risk assessment that is required prior to the commercial release of these crops. Of particular concern are non-target organisms (NTOs) that provide important ecosystem services. Here, we report on studies conducted in the Philippines over three cropping seasons with Bt eggplants expressing Cry1Ac for control of the eggplant fruit and shoot borer (EFSB), *Leucinodes orbonalis*, to examine potential effects on field abundance, community composition, structure and biodiversity of NTO’s, particularly non-target arthropod (NTA) communities. We document that many arthropod taxa are associated with Bt eggplants and their non-Bt comparators and that the number of taxa and their densities varied within season and across trials. However, we found few significant differences in seasonal mean densities of arthropod taxa between Bt and non-Bt eggplants. As expected, a lower abundance of lepidopteran pests was detected in Bt eggplants. Higher abundance of a few non-target herbivores was detected in non-Bt eggplants as were a few non-target beneficials that might control them. Principal Response Curve (PRC) analyses showed no statistically significant impact of Bt eggplants on overall arthropod communities through time in any season. Furthermore, we found no significant adverse impacts of Bt eggplants on species abundance, diversity and community dynamics, particularly for beneficial NTAs. These results support our previous studies documenting that Bt eggplants can effectively and selectively control the main pest of eggplant in Asia, the EFSB. The present study adds that it can do so without adverse effects on NTAs. Thus, Bt eggplants can be a foundational component for controlling EFSB in an Integrated Pest Management (IPM) program and dramatically reduce dependence on conventional insecticides.

## Introduction

Control of lepidopteran pests often relies on the use of broad spectrum insecticides which can negatively affect beneficial insect populations, often leading to pest resurgence, outbreaks of secondary pests, risk of off-farm movement of pesticides and environmental contamination [1–7]. An Integrated Pest Management (IPM) program for eggplant fruit and shoot borer (EFSB), *Leucinodes orbonalis*, the most damaging insect pest of eggplant (*Solanum melongena* L.) in South and Southeast Asia, has been proposed that would utilize resistant plant varieties, sex pheromones for trapping adults and disrupting mating, cultural controls such as removing infested plant parts and selective use of chemical insecticides [8]. Using resistant varieties, either developed through conventional breeding or genetic engineering means, should be the foundation of IPM [9]. However, conventional breeding has been unable to identify significant EFSB-resistance genes from cultivated eggplants and has not produced any commercial variety of eggplant conferring high level of resistance to the EFSB [10]. Furthermore, the cost of pheromones and labor-intensive cultural practices inhibits adoption of these pest management practices and so growers in Asia have become largely dependent on the frequent use of insecticides [11]. In the Philippines, farmers resort to frequent spraying (up to 72 times per 180 days cropping season) of mixtures of insecticides to control EFSB [12–16]. Broad-spectrum insecticides including profenofos, triazophos, chlorpyrifos, cypermethrin, and malathion are often used in eggplant production [14,17,18]. Such an insecticide-dependent strategy to control EFSB poses both environmental and health concerns.

Use of genetic engineering to develop insect-resistant plants offers a solution to the often-limited availability of highly insect-resistant germplasm [4,6,19,20]. Plants expressing insecticidal crystal (Cry) proteins from the bacterium *Bacillus thuringiensis* (Bt) have become a foundation for IPM [21] and were grown on 83.7 million ha globally in 2015 [22]. These crops have enabled more effective control of lepidopteran pests and led to increases in productivity while simultaneously reducing insecticide use and their associated negative environmental impacts [4, 23–26]. However, concerns have been raised that long term and extensive use of Bt crops could directly or indirectly affect biodiversity and beneficial non-target organisms, particularly arthropods [27–28]. Therefore, assessment of the environmental consequences of transgenic crops is an important prerequisite to their commercialization [29–32]. Risk of exposure to non-target arthropods (NTAs) by a Bt protein can be through direct feeding on plant tissues or consuming arthropods that have fed on plant tissues [23,31,33,34].

Agriculture depends on several arthropod groups performing ecological functions such as decomposition, pollination and biological control that are essential to soil health and crop productivity. This is especially true with eggplant, a crop producing lush growth over a long growing period, where high species diversity and interaction among and between herbivores and predators have been documented [35]. The eggplant non-target arthropod community includes predators, parasitoids, pollinators, sucking and chewing herbivores, and vagrant insects that are only temporary residents of the crop. Our studies included all these groups because it is important to understand how the dynamics of pests and beneficial species in eggplant fields may be affected so that the management practices can be adjusted as needed.

In the Philippines, field trials and an insect resistance management (IRM) plan are required prior to commercial release of insect-protected GM crops [36]. Data from field trials are needed to assess bioefficacy against the target pest and potential adverse effects on NTOs, particularly beneficial NTAs, and to formulate an appropriate IRM plan. The data presented in this report documents species abundance, diversity and community dynamics (composition and structure) of canopy-dwelling arthropods and soil micro-fauna in Bt and non-Bt eggplants in a study site located in Pangasinan, the largest eggplant growing province in the Philippines. These studies contain the first publicly available data on NTOs for Bt eggplants used to control *L*. *orbonalis*. The information generated here will contribute significantly to the theoretical and practical basis for environmental risk assessment of Bt eggplants in South and Southeast Asia.

## Materials and Methods

### Description of Trial Site

The studies were conducted at the same site in Bgy. Paitan, Sta. Maria, Pangasinan for three successive growing seasons from March 2010 to October 2012. The field trial site (15° 58' 35.07'' N, 120° 40' 33.62'' E), located in the province of Pangasinan, the Philippines, best represents the agro-climatic conditions and production practices of the largest eggplant growing region (Region I or the Ilocos Region) in the country. Based on the climate map of the Philippines [37], Pangasinan has Type 1 climate characterized by two pronounced seasons: dry, from November to April; wet, during the rest of the year. Farmers plant rice during the wet season. Eggplant cultivation in Pangasinan is primarily done during the dry season (DS). The province of Pangasinan has the largest production area (18.43%) and produces the largest volume of eggplants (31.95%) in the country (2005–2014 PSA data) [38]. Most importantly, the Pangasinan site represents the conditions that small-holder farmers are likely to experience relative to very high natural incidence of EFSB pressure that requires frequent insecticide applications.

### Plant materials

The NTO studies were conducted in the same Confined Field Trials (CFT) for Bt eggplant as described in Hautea *et al*. [39]. The experimental materials used in the series of three Confined Field Trial (CFT) experiments are listed in Table 1. Maharashtra Hybrid Seeds Co. Pvt. Ltd. (Mahyco) inserted the *cry1Ac* gene under the control of the constitutive 35S CaMV promoter into an eggplant elite line to control feeding damage caused by EFSB. The transformation event was designated as 'EE-1' [40,41]. The Bt eggplant lines (D2, D3, M1, M4, M8 used as test entries) in the field trials are advanced breeding lines (BC_3_F_4_ to BC_3_F_6_) derived from initial crosses of Mara selection x Mahyco EE-1 and DLP selection x Mahyco EE-1. The Cry1Ac protein levels expressed in the terminal leaves (shoots) of Bt eggplant lines ranged from 10.58–24.87 ppm dry weight, and with < 1% EFSB shoot damage compared with up to 46.6% shoot damage in non-Bt comparators [39].

**Table 1:** Plant materials used in confined field trials.

Trial No.	Crop Generation^1^	Duration^2^	Bt lines^3^	Non-Bt Counterparts ^4^	Non-Bt Commercial Variety ^5^
**1**	BC_3_F_4_	CY 2010	D2,D3	DLP	Mamburao
		(Mar- Jul 2010)	M1,M4,M8	Mara	
**2**	BC_3_F_5_	CY 2010–11	D2,D3	DLP	Mamburao
		(Sept 2010-Mar 2011)	M1,M4,M8	Mara	
**3**	BC_3_F_6_	CY 2012	D2	DLP	Mamburao
		(Mar-Oct 2012)	M1,M8	Mara S1,Mara S2	

^1^ BC_n_ = number of backcrossing; F_n_ = filial generation.

^2^ From sowing to end of fallow period.

^3^ Promising advanced Bt eggplant lines developed thru conventional backcross breeding; D2, D3 = Bt eggplant lines developed from Dumaguete Long Purple (DLP) x Mahyco event EE-1; M1, M4,M8 = Bt eggplant lines developed from Mara x Mahyco event EE-1.

^4^ DLP = improved line selection from public variety, DLP; Mara, Mara S1, Mara S2 = improved line selections from the cultivar Mara developed by UPLB-IPB Vegetable Breeding Division.

^5^National Seed Industry Council (NSIC)-registered commercial eggplant variety, ‘Mamburao’.

### Experimental design and field lay-out

Each field experiment was laid out in a randomized complete block design (RCBD) with four replications in each season. Each plot/entry consisted of 4 rows in Trial 1 and in Trial 2, and 6 rows in Trial 3. Each row had 10 plants. Planting distances were 1 m between rows and 0.75 m between plants. The perimeter of the field experiment was surrounded by five rows (1 m between rows) of conventional non-Bt eggplant varieties as pollen trap plants. A 200-meter radial distance isolated the field trial site from the nearest eggplants in the area. The field had been fallow for at least a year before it was used in the experiment. No plants were grown in the trial field until transplanting. Between trials, the field was fallow for at least 60 days before the next trial.

### Cultural and pest management

Seeds were sown in pots with sterilized soil and the seedlings were maintained inside the biosafety level 2 (BL2) greenhouse at UP Los Baños. At 28–30 days after sowing (DAS), representative seedlings of each entry were tested for presence or absence of Cry1Ac using immunoassay or a gene strip test kit, DesiGen Xpresstrip (DesiGen, Maharashtra, India), as described in Ripalda *et al*. [42]. Seedlings were transplanted in the field 30–34 DAS. The Confined Field Trials were managed based on the guidelines provided for the Vegetable National Cooperative Trial [43] and typical cultural practices for eggplant production in the area.

No insecticide sprays specific against eggplant fruit and shoot borer (EFSB) were applied during the growing period of the trials. Management of other arthropod pests and diseases was done by application of recommended IPM practices, primarily sanitation and witholding of pesticide use as long as possible to enable the proliferation of natural enemies. Only when it was necessary to reduce pest damage and preserve crop health, highly selective insecticides (i.e., thiamethoxam for leafhopper and whitefly and sulphur for mites) were applied. Spraying was always done after data collection and spray records were kept. All weeds were controlled regularly by manual weeding.

Permissions. All field trials were conducted according to the Biosafety Permit for Field Testing in Pangasinan (BPI Biosafety Permit No. 10-011b) issued by the Bureau of Plant Industry (BPI), Philippines on March 16, 2010. Prior to issuance of the field trial permit, the proposed trial site was inspected and approved by the BPI Post-Entry Quarantine Service (BPI-PEQS) office. The field inspection report on indicative conditions of the proposed field test site (BPI-FTI 001) contains information on the physical, biological and social environments of the site [44]. Required permission from the owner of the field trial site was also complied with. Various public participation activities (posting, municipal council meetings, public hearing, field visits, communications and outreach) were held before and through the duration of the field trials. All field trial activities were conducted under the supervision of the Institutional Biosafety Committee (IBC) and the BPI-PEQS office. During the conduct of the field trials, all biosafety conditions indicated in the Biosafety Permit were complied with. An IBC completion report was submitted at the end of the field trial period.

### Field sampling and species identification

#### Canopy-dwelling arthropods

Visual counts of non-target arthropods were taken from 16 plants (eight/row) from the two inner rows of each test plot/entry to minimize border effects. On each sample plant, easily visible and highly mobile non-target arthropods like spiders, coccinellids, and bees were counted without touching any plant part. Visual counting of minute arthropods was done by examining both surfaces of one young fully expanded leaf, one leaf near the middle of the canopy and one old leaf near the bottom of the canopy. For aerial predatory species like syrphid flies (*Parragus seratus*), the larvae, which are also plant canopy residents, were sampled assuming they would more likely be exposed to Bt protein than the adults. Sampling was conducted early in the morning (5:00–7:00 A.M.) when the field had not yet been disturbed by any field operations. Sampling weeks varied from 5 to 17 in each season. Whenever possible, common and frequently occurring arthropods were identified to species level. For less common species, identifications were made to family or order.

#### Soil microfauna

For minute ground-dwelling arthropods, one garden-trowel full of top soil, including litter and decaying debris, were collected from four randomly selected areas within the two inner rows (15 m^2^) of each plot/entry and pooled. Transparent plastic bags were used to hold the samples. From the pooled soil samples, 500 grams were taken and placed in a Berlese funnel 24 hours after bagging and brought to the Crop Protection Laboratory for extraction. The soil samples were subjected to 48 hour heat exposure using 80-watt incandescent bulb placed directly on top of the funnels. A small plastic bottle containing 50 ml of 70% ethanol was positioned at the bottom opening of each funnel to capture soil arthropods. Samples were collected two to three times throughout the eggplant growing season.

### Statistical Analysis

The mean abundance of individual NTAs in every test plot/entry per replication was computed. Then the mean abundance for Bt and non-Bt eggplants per replicate were computed by dividing the total number of individuals per taxa by the number of entries per crop type (Bt vs. non-Bt). For both trials 1 and 2, five Bt lines (D1, D3, M1, M4, M8) and two non-Bt near-isolines (DLP, Mara selections) plus the check (Mamburao) entries were considered. For trial 3, three Bt lines (D2, M1, M8) and three non-Bt near-isolines (DLP, Mara S1, Mara S2) were used.

All arthropod species found in Bt and non-Bt eggplants were classified, grouped and recorded into the following functional guilds: predators, herbivores or non-target pests, parasitoids, pollinators and vagrants. Non-target herbivores or pests were further classified into sucking and chewing arthropods. Vagrants refer specifically to those insects, including accidental visitors, with no clear association with eggplant (e.g. herbivores or pests from other plants in surrounding areas, or adults whose immatures are saprophytes or living in aquatic environment). The composition and relative proportion between each guild were calculated. Differences in the composition of taxa among functional guilds and taxa within guilds in Bt and non-Bt eggplants were analyzed using Mann-Whitney U-test in PROC NPAR1WAY in SAS [45]. Based on the Wilcoxon statistic, normal approximation with two-sided p-value was used at 5% level of significance.

#### Univariate Analysis

Analyses on seasonal mean NTAs abundance were carried out using a mixed model, repeated measures ANOVA in PROC MIXED in SAS [45], with block as a random effect, week as repeated measure and eggplant type (Bt and non-Bt) as a fixed effect. An autoregressive heterogenous (ARH1) covariance structure was modelled. Separate analyses were conducted for each season. Differences in LSMEANS were used to test for differences in abundance between Bt and non-Bt eggplants for each sampling date for each taxon. NTAs abundance data were log transformed (log [x + 1]) prior to analysis to meet the assumptions for normality and homogeneity of residuals, but untransformed means are presented.

#### Principal Response Curve (PRC) analysis

The effect of Bt eggplants on the community of non-target arthropods was evaluated by principal response curve (PRC) analysis using CANOCO for Windows v4.56 [46]. PRC is a multivariate ordination method designed to test and display treatment effects, relative to a standard (here non-Bt eggplant), that change across time [23, 47]. To test whether crop type was significant a Monte Carlo permutation test (499 permutations, restricted for split plot design) on the first canonical axis of the RDA was conducted [48]. This process permutes within treatment plots but does not permute across time [23] NTA abundance data were log-transformed to reduce the effect of weights inflated because of highly abundant species [33]. Crop type was considered as environmental variable, blocks and sampling weeks were defined as co-variables and the interaction of crop type and sampling weeks as explanatory variable.

#### Diversity index

The Shannon-Wiener index [49] was used to measure diversity and evenness of non-target arthropods. A diversity index provides more information about community composition than simply species richness. The Shannon-Wiener index takes the relative abundances of different species into account thus providing information about rarity and commonness of species in a community. The Shannon-Wiener diversity (*H’*) and Shannon’s equitability (*E*) indices were calculated. Shannon indices for non-target arthropods for each week were compared for each season using repeated measure ANOVA in SAS [45].

#### Rank abundance

Rank abundance diagrams were constructed by plotting the relative abundances of species against their rank in the samples [50]. The outlines of this diagram characterize the structures of non-target arthropod communities in Bt and non-Bt eggplants. Spearman rank correlation coefficient (*r*) was computed to measure the strength of linear relationship of rank abundances of non-target arthropods between crop types, with two significance levels: *P* = 0.05 and *P* = 0.01. Spearman rank correlation was calculated using the PROC CORR procedure in SAS [45].

Data are available from the Dryad Digital repository: http://dx.doi.org/10.5061/dryad.6c8s6 [51]

## Results

### NTA abundance in Bt and non-Bt eggplants

A total of 91 taxa were observed in Bt and non-Bt eggplants during the three-season duration of the study. The full lists of arthropods observed per trial, classified according to functional guilds, and results of univariate analyses of their seasonal mean abundance are presented (S1 Table). There were more taxa observed during the dry season (Trial 2), which is the main planting season for eggplant in Pangasinan, than during the wet/off-season trials (Trials 1 and 3). No significant differences in the seasonal mean abundance were detected in 81.3% (84/91) of the total NTAs observed between Bt and non-Bt eggplants. Significant differences were observed in some hemipterans (jumping plant bug (*Halticus minutus*), leafhoppers (*Amrasca biguttula*), mirid bugs (*Campylomma* sp., *Cyrtopeltis* sp.), whitefly (*Bemisia tabaci*)), non-target lepidopterans (leaf folder (*Homona coffearia*), lepidopteran leafminer (*Phycita* sp.), semilooper (*Chrysodeixis eriosoma*), tomato fruitworm (*Helicoverpa armigera*) and coccinelids (Coccinelidae). Of these taxa that showed significant differences in seasonal mean abundance, the differences were observed only in one or two weeks out of the 5 to 17-week sampling periods each season (S1 Fig). Furthermore, some of these species were not consistently detected in every trial, and some were associated alternately with either Bt or non-Bt eggplants.

### Composition of NTA communities in Bt and non-Bt eggplants

**Fig 1:**
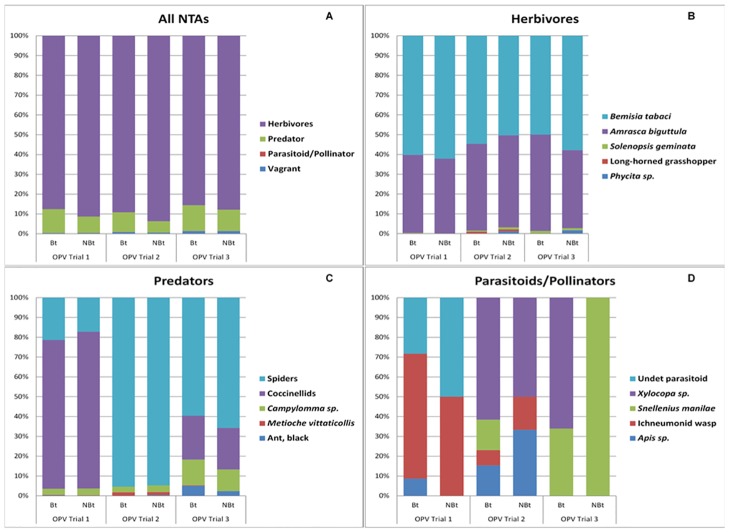
Composition of non-target arthropod (NTA) communities in Bt and non-Bt eggplants. (a) All (or Total) NTAs communities; (b) Herbivores; (c) Predators; (d) Parasitoids and Pollinators

The eggplant arthropod community recorded in Bt and non-Bt eggplants consisted of herbivores or non-target pests, predators, parasitoids and pollinators, and vagrant insects (Fig 1). Herbivores were by far the most abundant guild, followed by predators while parasitoids and pollinators were rare. Among the different functional guilds, significant differences were only detected in the herbivore guild between Bt and non-Bt eggplants in every trial (Table 2). Analyses of the distribution of the species within guilds confirmed that the most abundant taxa observed were mostly the ones also detected to have significant differences in seasonal mean densities (S1 Table). Among the herbivores (Fig 1b) whiteflies (*B*. *tabaci*) and leafhoppers (*A*. *biguttula*) were the most abundant in all three trials and spiders (Araneae) and coccinelids (Coccinellidae) were the most abundant among the predators (Fig 1c). Among the parasitoids and pollinators (Fig 1d), an ichneumonid wasp (Ichneumonidae) and honeybee (*Apis* sp.) were common in trials 1 and 2, while a cutworm parasitoid (*Snellenius manilae*) and honeybee (*Apis* sp.) were common in trials 2 and 3. The composition of vagrant species was not presented because these are mostly occasional arthropod visitors from surrounding plants, which have no clear association with eggplant.

**Table 2:** Mean comparison of NTA abundance among functional guilds and taxa in Bt and non-Bt eggplants.

Guild/Taxa	Trial 1			Trial 2			Trial 3		
	Bt	Non-Bt	*P*	Bt	Non-Bt	*P*	Bt	Non-Bt	*P*
**Herbivores**	131.671	240.069	< .0001	105.044	194.304	< .0001	98.717	138.683	0.001
*Bemisia tabaci*	78.538	148.174	< .0001	56.062	96.539	< .0001	48.267	78.583	0.001
*Amrasca biguttula*	51.458	89.979	< .0001	45.068	89.289	0.0335	46.967	53.600	0.747
*Solenopsis geminata*	0.429	0.361	0.8214	0.524	2.039	< .0001	1.217	1.383	0.915
Tettigonidae	0.029	0.021	0.6213	0.991	1.848	0.0004	0.083	0.017	0.172
*Phycita* sp.	-	-	-	0.026	2.201	< .0001	0.000	2.317	< .0001
**Predators**	18.121	21.708	0.1403	25.297	26.049	0.4896	14.917	17.017	0.175
Araneae	3.842	3.694	0.7890	10.847	10.971	0.9424	8.783	11.050	0.003
Coccinellidae	13.438	16.972	0.1606	1.021	1.162	0.1980	3.267	3.533	0.950
*Campylomma* sp.	0.550	0.722	0.0132	0.318	0.397	0.2672	1.917	1.833	0.690
*Metioche vittaticollis*	0.033	0.035	0.7731	0.185	0.167	0.8536	0.033	0.000	0.159
Formicidae	0.058	0.042	0.6095	0.018	0.049	0.0709	0.750	0.400	0.547
**Parasitoids and Pollinators**	0.050	0.063	0.6661	0.053	0.034	0.2524	0.050	0.050	1.000
Undetermined parasitoid (Hymenoptera)	0.013	0.028	0.5215	-	-	-	-	-	-
*Snellenius manilae*	-	-	-	0.006	0.000	0.2741	0.017	0.050	0.560
Ichneumonid wasp	0.029	0.028	0.5273	0.003	0.005	0.7168	-	-	-
*Xylocopa* sp.	-	-	-	0.024	0.015	0.3441	0.033	0.000	0.325
*Apis* sp.	0.004	0.000	0.4418	0.006	0.010	0.6059	-	-	-
**Vagrants**	0.538	1.118	0.0027	1.112	1.221	0.3207	1.600	2.167	0.287

### NTA community dynamics in Bt and non-Bt eggplants

**Fig 2:**
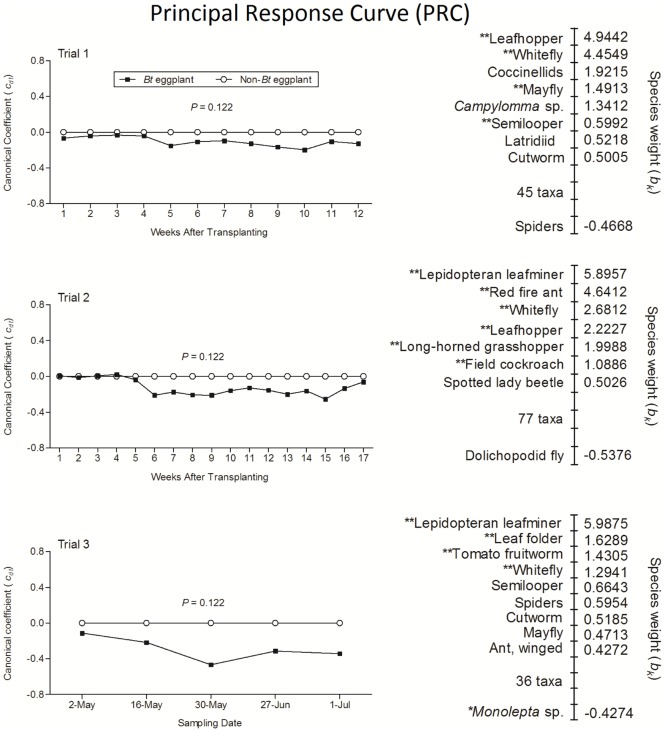
Principal response curve patterns and species weight of non-target arthropod (NTA) communities in Bt and non-Bt eggplants. Vertical axis represents the difference in community structure between Bt and non-Bt eggplants expressed as regression coefficient (*Cdt*) of the PRC model. The *P* value indicates significance of the PRC over time based on restricted Monte Carlo permutation test. The species weight (*b*_*k*_) can be regarded as affinity of the taxon to the principal response. Only species with weights less than -0.05 or greater than 0.5 are shown.

The Principal Response Curve (PRC) analyses of NTA abundance data in the three trials revealed no significant difference between Bt and non-Bt eggplants (Fig 2). A large proportion of the total variance was explained by sampling weeks and only a small portion was attributed to crop type (Bt vs. non-Bt) in the first axis of the redundancy analysis (Table 3). Analyses of the distribution of the species weight (*b*_*k*_) confirmed that the taxa with high species weight were the same ones with significant differences in seasonal mean densities detected by univariate analysis (S1 Table). The most abundant species in the NTAs communities detected in Bt and non-Bt eggplants were lepidopteran leafminer (*Phycita* sp), leafhopper (*A*. *biguttula*), whitefly (*B*. *tabaci*), red fire ant (*S*. *geminata*), *Phaneroptera* sp., tomato fruit worm (*H*. *armigera*), mayfly (Ephemenoptera), semilooper (*C*. *eriosoma*), field cockroach (Blatellidae), coccinelids (Coccinelidae), *Campylomma* sp., latridiid (Latridiidae), cutworm (*Spodoptera litura*), spotted lady beetle (*Epilachna* spp.), winged ant (Formicidae) and spider (Araneae), dolichopodid fly (Dolichopodidae), *Monolepta* sp. Species with weights between -0.5 and 0.5 are not shown because they are likely to show a weak response or a response that is unrelated to the principal response curve [48].

**Table 3:** Characteristics of Principal Response Curves (PRC) for non-target arthropod communities in Bt and non-Bt eggplants.

Parameters	Statistics^1^		
	Trial 1	Trial 2	Trial 3
***F* value**	2.094	3.724	3.479
***P* value (calculated using Monte Carlo simulation, 499 permutations)**	0.122	0.122	0.122
**Variance explained by crop type**	7.6	13.5	15.9
**Proportion of this variance captured by PRC**^1^	61.7	57.7	64.9
**Variance explained by sampling date**	28.6	34.5	40.7

^1^Values in the table were generated by Principal Response Curve analyses of log (x + 1) transformed non-target arthropod abundance data

### Other descriptors of NTA communities

**Fig 3:**
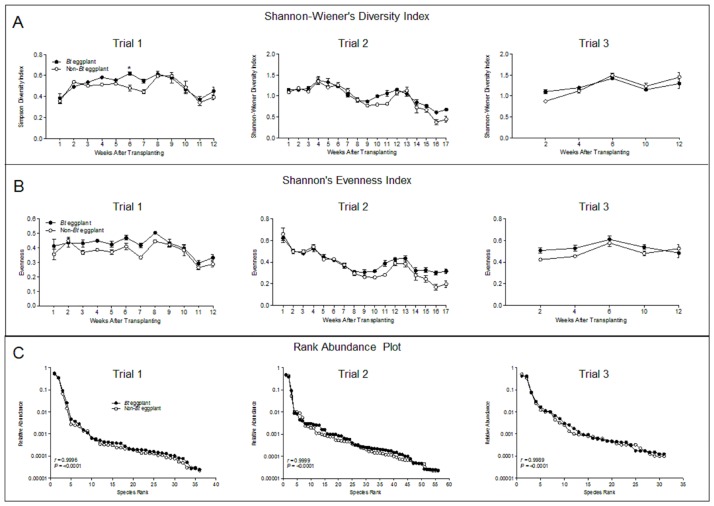
Other descriptors of non-target arthropod (NTA) communities in Bt and non-Bt eggplants. (A) Shannon’s diversity index and (B) Shannon’s evenness index; *P* value indicates significance between indices (not significant, *P*> 0.05). (B) Rank abundance plots; Spearman rank correlation coefficient (*r*) indicates a very strong positive correlation between Bt and non-Bt eggplants. Y axis is log_10_ scale.

Diversity, indicated by species richness and evenness was also monitored using two descriptors of NTA community structure: Shannon diversity index and evenness [49] and rank abundance curves [50]. There were no significant differences in the Shannon diversity index (Fig 3A) and evenness (Fig 3B) of NTA communities between Bt and non-Bt eggplants for either measure (*P*>0.05). Temporal changes in mean values of the diversity and evenness indices also showed no significant differences between the NTAs communities in Bt and non-Bt eggplants except in only one out of the 12-week sampling periods (week 6) in trial 1 of the Shannon diversity index (Fig 3A). Spearman rank correlation coefficient (*r* = 0.99) indicates very strong positive correlation between rank abundances of NTA communities in Bt and non-Bt eggplants (Fig 3B). The first and second ranked species, represented by whiteflies and leafhoppers, were consistently the most dominant species in both Bt and non-Bt eggplants. This was evident in the sudden decline to the third ranked species (Fig 3B).

### Abundance of soil-dwelling arthropods

**Fig 4:**
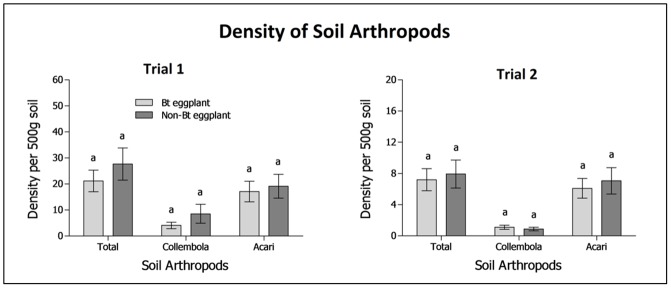
Species density of arthropods in the soil planted to Bt and non-Bt eggplants. Trials 1 and 2, CY 2010–2011. Common letters above the bars indicate no significant differences among the densities. Note different scales on y-axis.

No statistically significant differences were observed in the mean density of collembolans and mites between Bt and non-Bt eggplants (Fig 4).

## Discussion

Numerous studies, reviews and meta-analyses have assessed the impacts of Bt cotton and maize on non-target organisms, in particular non-target arthropods (NTAs) [1–7, 25, 29–32, 52–58]. Limited work has examined the impact of Bt eggplants producing coleopteran-active Cry3Bb [59,60], but to our knowledge this is the first report of a field study that assessed the impact of Bt eggplants expressing Cry1Ac on NTAs and other organisms. This study helps to address concerns on the potential environmental risks to NTAs of Bt eggplant cultivation in the Philippines and similar areas. Herein, we monitored the abundance of canopy- and soil-dwelling NTAs in Bt and non-Bt eggplants for three seasons over 2.5 years at a field trial site located in Pangasinan, the largest eggplant growing area in the country.

We found no significant impact of Bt eggplants on the abundance of most canopy-dwelling NTAs. Seasonal mean abundance of more than 80% of the taxa observed in Bt and non-Bt eggplants were similar. Of taxa that showed significant differences in seasonal mean abundance between Bt and non-Bt eggplants, the differences were detected over time indicating that changes in NTAs were driven more by temporal dynamics rather than crop type. Some species were alternately associated with either Bt or non-Bt eggplants suggesting normal species variation seen in agricultural fields and not associated with the experimental treatments. The preference of some of the NTAs could have been affected by the difference observed in a few morphological traits (e.g. leaf shape, size, lateral branches) between Bt and non-Bt eggplants. Although the two crop types have related genetic backgrounds, the observed difference in leaf type (broad and narrow) in the non-Bt cultivar Mara, which was a selection from a farmer’s variety, could be attributed to the inherent heterogeneity in open-pollinated autogamous species [61]. The backcross breeding that developed the Bt eggplant lines derived from Mara only selected for the narrow-leaf type characteristic of the Mara recurrent parent. It is likely that the damage caused by EFSB in the non-Bt eggplants resulted in production of more lateral branches due to the suppression of apical dominance [62]. Overall, these findings suggest that Bt eggplant did not adversely affect species abundance in the NTA community.

The analysis of functional guilds revealed that the composition of common and rare guilds are similar in Bt and non-Bt eggplants except in the herbivore guild, but that differences were attributed only to a few species, mostly lepidopteran non-target pests and hemipterans. PRC analysis revealed no significant impact of Bt eggplants on NTA communities through the growing season when compared to non-Bt eggplants in all three trials. The large proportion of the total variance was accounted for by sampling weeks and much less by crop type, indicating that changes in abundance of NTAs was driven by time rather than due to exposure to Bt eggplant expressing the Cry1Ac insecticidal protein. Finally, we found little difference in the diversity, evenness and rank abundance of NTA communities in Bt and non-Bt eggplants. If Bt eggplants had a negative impact, we would have expected lower species richness and evenness in comparison to non-Bt eggplants. Previous studies on Bt cotton expressing Cry1Ac or Bt maize expressing Cry1Ab found similar results [25,30,34,58,63, 64].

Mites (Acari) and collembolans have been used as representative soil invertebrates for monitoring the environmental impacts of transgenic plants [57,65, 66]. Here, we found no differences in abundance of these taxa between Bt and non-Bt eggplants. This is consistent with previous work on long-term cultivation of Bt cotton (Cry1Ac), which showed no significant effect on the abundance of soil invertebrates including collembolans, mites and spiders [57, 58]. Similar results were also observed in Bt maize (Cry1Ab) where activity and abundances of ground-dwelling invertebrates, spiders, carabid and rove beetles, did not differ in Bt crops compared with near-isogenic control plots [33, 65–67].

Herbivores and predators were the most abundant functional guilds found in Bt and non-Bt eggplants. Among the herbivores, hemipterans and secondary lepidopteran pests were the most abundant species. As expected, significantly lower abundance of secondary lepidopteran pests was detected on Bt eggplants compared with non-Bt eggplants because Cry1Ac expressed in Bt eggplants is known to be efficacious against many Lepidoptera and the trials were not sprayed with lepidopteran-specific insecticides. The two most abundant sucking insect pests, leafhopper (*A*. *biguttula*) and whitefly (*B*. *tabaci*), had lower abundance in Bt compared with non-Bt eggplants. This result is consistent with previous reports that showed decreases in abundance of some hemipterans, including cicadellids or leafhoppers, on Bt cotton compared to those on non-Bt cotton [23,33]. Mechanisms causing such difference could be varied, and one such study demonstrated that herbivore-induced plant compounds can affect a secondary pest (6). A meta-analysis of effects of Bt crops on NTOs [55] also showed that when fields of insecticide-free Bt crops were compared with insecticide-free control fields, certain non-target taxa were less abundant in Bt fields, including coleopterans and hemipterans in Bt cotton, and hymenopterans in Bt maize. This latter effect was due entirely to the expected reductions in a specialist parasitoid of the main lepidopteran target of Bt maize [56]. In contrast, many studies have shown that Bt cotton producing Cry1Ac did not affect the densities of many non-lepidopterans including leafhopper and whitefly [68–72]. A possible explanation for the higher abundance of leafhopper and whitefly observed in non-Bt eggplants in the present study was the production of more lateral branches in non-Bt eggplants (M. Navasero, personal observation) resulting from damage in the terminal shoots caused by the primary target pest, EFSB. Suppression of the apical dominance of the plant likely induced more lateral bud outgrowth, giving rise to lateral branches [62]. The resulting dense canopy may have provided a more favorable microclimate conducive to growth and multiplication of these pests.

In the case of predators, the most abundant were coccinelids and spiders. Coccinelids showed significantly higher abundance in non-Bt than in Bt eggplants and this was likely the result of higher prey abundance in the non-Bt eggplants. The prey consisted not only of lepidopterans, but the higher abundance of leafhoppers and whiteflies. Our findings are consistent with previous reports on Bt cotton where reduced number of prey, particularly of lepidopterans [73] and sucking insect pests [23,33] were observed. Our results also agree with previous research syntheses [25,56] in which the abundance of members of the predatory arthropod guild were slightly reduced in unsprayed Bt cotton expressing Cry1Ac compared to the unsprayed non-Bt control. This pattern was driven by the abundance of very few taxa, but the consequences of such reductions likely do not significantly affect the biological control services provided by the predator community overall [70,74].

In conclusion, our non-target studies of Bt eggplants over three growing seasons in the largest eggplant production province of the Philippiines with the highest EFSB pest pressure showed that arthropod communities, except for the target pest species, would be largely unaffected by the cultivation of this new crop. We reported previously that Bt eggplant demonstrated nearly 100% control of its major pest, EFSB, without the use of supplemental sprays [39]. Ex-ante studies for Bt eggplant in the Philippines [12, 13] indicated that producers and consumers would be benefited by Bt eggplant technology adoption. At the farm level, Bt eggplant adoption has high potential to increase marketable yield, reduce costs, and increase profits. Farmers would gain profits because the technology would reduce EFSB damage, increase the marketable yield and lower production costs. Consumers would have an adequate supply of safer eggplant at a lower price. The adoption of Bt eggplant is projected to greatly reduce pesticide use on eggplant, thereby reducing both pesticide loading in the environment and hazards to farm laborers and consumers. Bt eggplant presents a more efficacious, environmentally benign and profitable alternative to the current practice of intense use of chemical insecticides in eggplant production.

## Supporting Information

S1 Fig Temporal occurrence and seasonal mean density of non-target arthropod (NTA) with significant differences in Bt vs. non-Bt eggplants. (a) Trial 1. (b) Trial 2. (c) Trial 3. Arrows indicate the week wherein the difference in density is statistically significant between crop types (*P*< 0.05). Note different scales on y axes.
(TIF)

S1 Table Seasonal mean ± SEM abundance of NTAs in Bt and non-Bt eggplants. (a) herbivorous sucking and chewing insects; (b) predatory arthropods; (c) parasitoids and pollinators; (d) vagrant insects.
(DOCX)
