# Supplementary figures and images for: Assessing Potential Impact of Bt Eggplants on Non-Target Arthropods in the Philippines
Source: PLoS One. 2016 Oct 31;11(10):e0165190. doi: 10.1371/journal.pone.0165190 (PMC5087897; doi:10.1371/journal.pone.0165190)

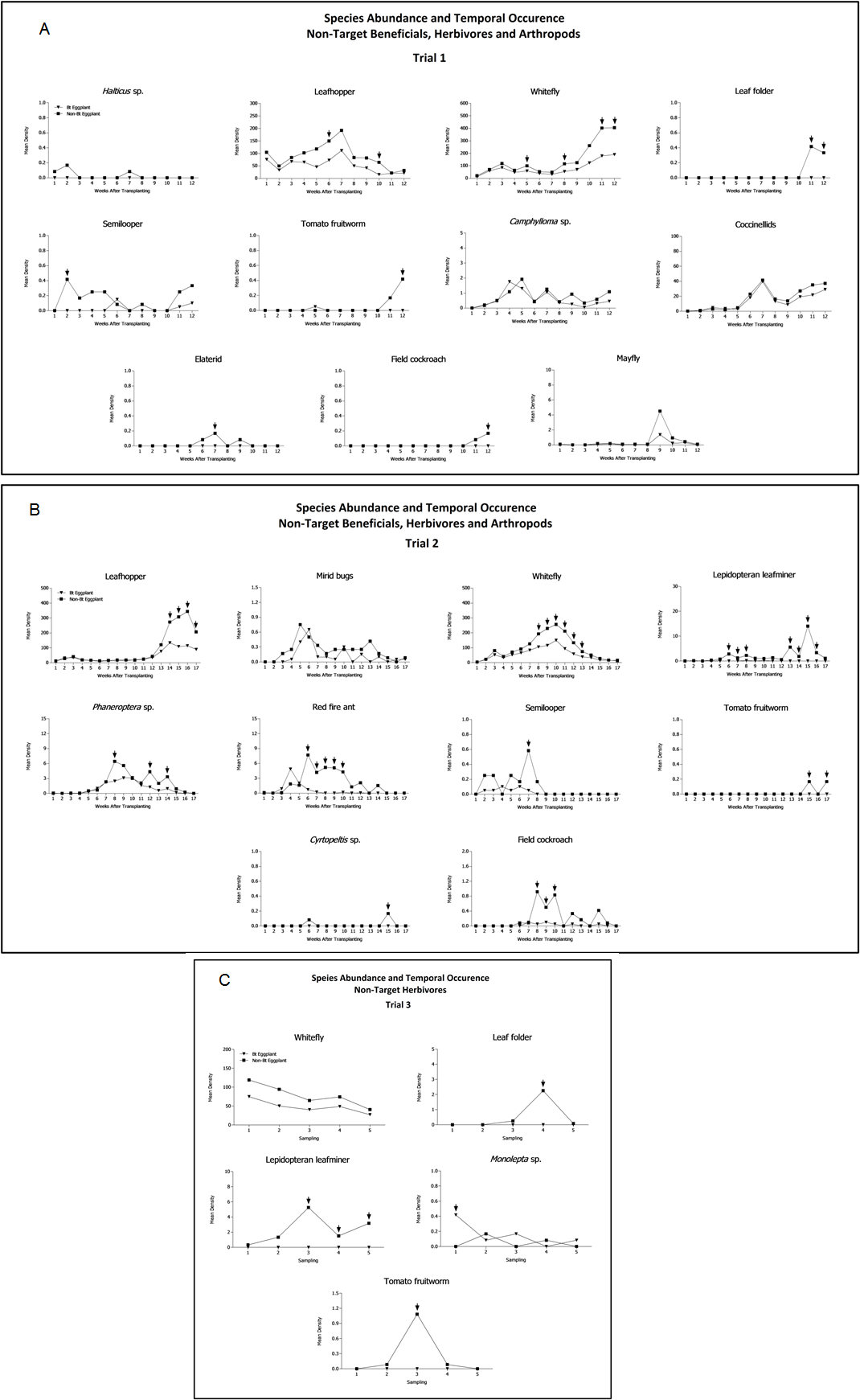

Supplement: S1 Fig — (a) Trial 1. (b) Trial 2. (c) Trial 3. Arrows indicate the week wherein the difference in density is statistically significant between crop types (P< 0.05). Note different scales on y axes. (TIF) [file pone.0165190.s001.tif]
