# Supplementary material for: Assessing Potential Impact of Bt Eggplants on Non-Target Arthropods in the Philippines
Source: PLoS One. 2016 Oct 31;11(10):e0165190. doi: 10.1371/journal.pone.0165190 (PMC5087897; doi:10.1371/journal.pone.0165190)
Supplement: S1 Table — (a) herbivorous sucking and chewing insects; (b) predatory arthropods; (c) parasitoids and pollinators; (d) vagrant insects. (DOCX) [file pone.0165190.s002.docx]

**S1 Table**. **Seasonal mean ± SEM abundance of NTAs in Bt and non-Bt eggplants.** (a) Herbivorous sucking and chewing insects observed by visual samplings on Bt and non-Bt eggplants. Bgy. Paitan, Sta. Maria, Pangasinan, Philippines. Trials 1-3, 2010-2012.

^1^herbivorous chewing pests

^2^consists of several taxa

^3^statistics from repeated measures ANOVA of log (x+1) transformed data observed at weekly interval on Bt eggplant lines containing event 'EE-1) and conventional non-Bt eggplant comparators (**P*< 0.05, ** *P*< 0.01)^4^ no data

**S1 Table**. **Seasonal mean ± SEM abundance of NTAs in Bt and non-Bt eggplants.** (b) Predatory arthropods observed by visual samplings on Bt vs. non-Bt eggplants Bgy. Paitan, Sta. Maria, Pangasinan, Philippines, Trials 1-3, 2010-2012.

^1^beneficial arthropods

^2^consists of several taxa

^3^statistics from repeated measures ANOVA of log (x+1) transformed data observed at weekly interval on Bt eggplant lines containing event 'EE-1) and conventional non-Bt eggplant comparators (**P*< 0.05, ** *P*< 0.01)

^4^no data

**S1 Table.**  **Seasonal mean ± SEM abundance of NTAs in Bt and non-Bt eggplants.** (c) Parasitoids and pollinators observed by visual samplings on Bt vs. non-Bt eggplants. Bgy. Paitan, Sta. Maria, Pangasinan, Philippines. Trials 1-3. 2010-2012.

^1^beneficial arthropods

^2^consists of several taxa

^3^statistics from repeated measures ANOVA of log (x+1) transformed data observed at weekly interval on Bt eggplant lines containing event 'EE-1) and conventional non-Bt eggplant comparators (**P*< 0.05, ** *P*< 0.01)

^4^no data

**S1 Table.** **Seasonal mean ± SEM abundance of NTAs in Bt and non-Bt eggplants.** (d) Vagrant insects observed by visual samplings on Bt vs. non-Bt eggplants. Bgy. Paitan, Sta. Maria, Pangasinan, Philippines, Trials 1-3, 2010-2012.

^1^other arthropods not directly associated with eggplant

^2^statistics from repeated measures ANOVA of log (x+1) transformed data observed at weekly interval on Bt eggplant lines containing event 'EE-1 and conventional non-Bt eggplant comparators (**P*< 0.05, ** *P*< 0.01)

^3^no data
